# Supplementary material for: The Changing Detection Rate of Respiratory Syncytial Virus in Adults in Western Australia between 2017 and 2023
Source: Viruses. 2024 Apr 23;16(5):656. doi: 10.3390/v16050656 (PMC11125702; doi:10.3390/v16050656)
Supplement: Supplementary file 1 [file viruses-16-00656-s001.zip › viruses-2962772-supplementary.pdf]

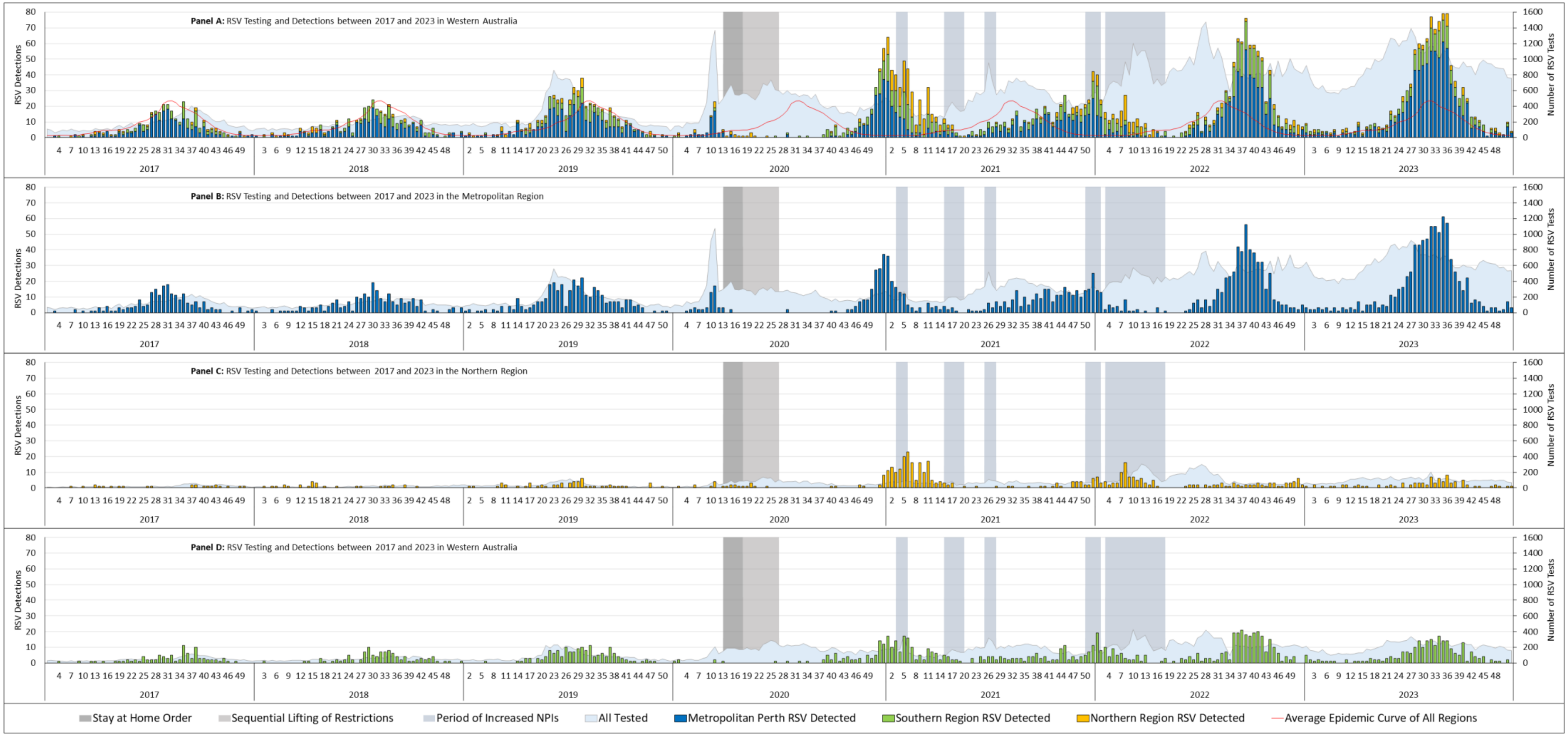

Figure S1. RSV testing and detections per week by region between 2019 and 2021, with the associated SARS-CoV-2 related non-pharmaceutical interventions in all regions (panel A), in Metropolitan Perth (panel B) and Northern Region (panel C) and Southern Region (panel D). The average epidemic curve was calculated using RSV detection data between 2012-2019. The average epidemic curve was calculated using RSV detection data between 2012-2019. Overview of non-pharmaceutical interventions: the first local case of SARS-CoV-2 was in week eight of 2020. State-wide stay-at-home order (weeks 14–17 of 2020) was followed by sequential lifting of restrictions (18–26 weeks), with gatherings of 10 allowed at week

18, 20 at 21 weeks and 100 at week 24. There were periods of increased NPIs that included mask requirements in the Metropolitan and Southern regions; weeks 4 and 5, weeks 16 to 19 and 26 to 27 in 2021 and week 50 in 2021 to week 2 in 2022 and week 3 to 17 in 2022. Borders restrictions were fully removed in week 9 of 2022. There was sporadic community detection of SARS-CoV-2 during the periods of increased NPIs in 2022. Continued low-level local transmission of SARS-CoV-2 was observed in January 2022, exceeding one hundred local cases per day by week 6 of 2022. RSV, respiratory syncytial virus.

Table S1. Detection rate per 100,000 per season between 2017 and 2023. 95% CI, 95% confidence interval; RSV, respiratory syncytial virus.

Seasons	2017	2018	2019	2020/21	2021/22	2022	2023
	Seasonal Detection Rate (95% CI)	Seasonal Detection Rate (95% CI)	Seasonal Detection Rate (95% CI)	Seasonal Detection Rate (95% CI)	Seasonal Detection Rate (95% CI)	Seasonal Detection Rate (95% CI)	Seasonal Detection Rate (95% CI)
All	13.9 (12.3 to 15.6)	16.6 (14.9 to 18.5)	22.9 (20.9 to 25.1)	29.5 (27.3 to 31.9)	24.5 (22.5 to 26.7)	31.1 (28.8 to 33.5)	41.6 (39 to 44.3)
By Region							
Metropolitan Perth	11 (9.5 to 12.7)	12.4 (10.8 to 14.2)	16.9 (15 to 19)	15.7 (13.8 to 17.7)	17.6 (15.7 to 20)	25.6 (23.3 to 28.1)	40 (37 to 43)
Northern Region	20.6 (11.2 to 34.5)	32.3 (20.2 to 48.9)	61.7 (44.4 to 83.3)	267.2 (229.8 to 309)	116 (92 to 144.5)	47 (32.1 to 66.3)	89.6 (68.5 to 115)
Southern Region	22.6 (18 to 28.2)	25.8 (20.8 to 31.6)	40.6 (34.3 to 47.8)	50.1 (43.1 to 58.1)	43.4 (36.9 to 50.8)	57.4 (49.8 to 65.9)	51.3 (44.1 to 59.3)

Table S2. The average baseline seasonal detection rate per 100,000 between 2017 and 2019 compared to the detection rate of the seasons observed between 1st January 2020 and 31st December 2023. The detection rate ratio was calculated using the detection rate of the average season between 2017 to 2019 compared to the individual observed seasons. * Number rounded up to 1. 95% CI, 95% confidence interval; DRR, Detection Rate Ratio. RSV, respiratory syncytial virus.

	2017 to 2019 Baseline Season	2020/21	2021/22	2022	2023
	Seasonal Detection Rate (95% CI)	Seasonal Detection Rate Ratio (95% CI)	Seasonal Detection Rate Ratio (95% CI)	Seasonal Detection Rate Ratio (95% CI)	Seasonal Detection Rate Ratio (95% CI)
All	17.9 (16.1 to 19.8)	DRR 1.7 (1.5 to 1.9)	DRR 1.4 (1.2 to 1.6)	DRR 1.7 (1.5 to 2)	DRR 2.3 (2.1 to 2.6)
By Region	Seasonal Detection (95% CI)	Seasonal DRR (95% CI)	Seasonal DRR (95% CI)	Seasonal DRR (95% CI)	Seasonal DRR (95% CI)
Metropolitan Perth	13.4 (11.7 to 15.3)	DRR 1.2 (1* to 1.4)	DRR 1.3 (1.1 to 1.6)	DRR 1.9 (1.6 to 2.2)	DRR 3 (2.6 to 3.5)
Northern Region	38.2 (24.9 to 55.9)	DRR 7 (4.6 to 11)	DRR 3 (1.9 to 4.9)	DRR 1.2 (0.7 to 2.2)	DRR 2.3 (1.5 to 3.9)
Southern Region	29.7 (24.3 to 35.9)	DRR 1.6 (1.3 to 2.1)	DRR 1.4 (1.1 to 1.8)	DRR 1.9 (1.5 to 2.5)	DRR 1.7 (1.4 to 2.2)

Table S3. RSV detections per year and the proportion of total detections by region and age group. 95% CI, 95% confidence interval; RSV, respiratory syncytial virus. * = RSV season disrupted during this period.

Year	2017	2018	2019	2020*	2021*	2022*	2023
Detection By Region	n (% of Total)	n (% of Total)	n (% of Total)	n (% of Total)	n (% of Total)	n (% of Total)	n (% of Total)
Metropolitan Perth	209 (64)	231 (63)	335 (61)	182 (63)	422 (47)	562 (54)	787 (71)
Northern Region	24 (7)	28 (8)	56 (10)	31 (11)	214 (24)	147 (14)	87 (8)
Southern Region	92 (28)	106 (29)	158 (29)	76 (26)	256 (29)	328 (32)	238 (21)
Detections By Age Groups	n (% of Total)	n (% of Total)	n (% of Total)	n (% of Total)	n (% of Total)	n (% of Total)	n (% of Total)
≥16 to ≤39 years	58 (17)	53 (13)	108 (19)	93 (32)	278 (30)	291 (27)	211 (18)
≥40 to ≤ 59 years	75 (23)	101 (25)	139 (25)	74 (25)	230 (25)	262 (24)	219 (19)
≥60 to ≤74 years	107 (32)	119 (30)	129 (23)	65 (22)	181 (20)	260 (24)	324 (28)
≥75 years	93 (28)	127 (32)	186 (33)	62 (21)	230 (25)	264 (25)	404 (35)

Table S4. The average RSV detection rate per 100,000 per year of the 2017 to 2019 baseline period compared to the detection rate per year between 2020 and 2023. The detection rate ratio was calculated using the average detection rate of the 2017 to 2019 baseline period compared to the individual observed years. 95% CI, 95% confidence interval; DRR, Detection Rate Ratio. RSV, respiratory syncytial virus

	2017 to 2019 Baseline	2020	2021	2022	2023
	Detection Rate/Year (95% CI)	DRR (95% CI)	DRR (95% CI)	DRR (95% CI)	DRR (95% CI)
All	20.8 (18.9 to 22.9)	DRR 0.7 (0.6 to 0.8)	DRR 2 (1.8 to 2.3)	DRR 2.3 (2.1 to 2.6)	DRR 2.4 (2.2 to 2.7)
By Region	Detection Rate/Year (95% CI)	DRR (95% CI)	DRR (95% CI)	DRR (95% CI)	DRR (95% CI)
Metropolitan Perth	15.3 (13.5 to 17.2)	DRR 0.7 (0.6 to 0.9)	DRR 1.6 (1.4 to 1.9)	DRR 2.2 (1.9 to 2.5)	DRR 3.1 (2.6 to 3.5)
Northern Region	52.8 (37 to 73.2)	DRR 0.9 (0.5 to 1.4)	DRR 5.9 (4.2 to 8.7)	DRR 4.1 (2.8 to 6.1)	DRR 2.4 (1.6 to 3.7)
Southern Region	33.3 (27.6 to 39.9)	DRR 0.6 (0.5 to 0.9)	DRR 2.2 (1.7 to 2.7)	DRR 2.8 (2.2 to 3.4)	DRR 2 (1.6 to 2.5)
By Age Group	Detection Rate/Year (95% CI)	DRR (95% CI)	DRR (95% CI)	DRR (95% CI)	DRR (95% CI)
≥16 to ≤39 years	8.3 (6.5 to 10.4)	DRR 1.3 (0.9 to 1.7)	DRR 3.8 (2.9 to 4.9)	DRR 3.9 (3 to 5.1)	DRR 2.7 (2.1 to 3.6)
≥40 to ≤ 59 years	15.5 (12.7 to 18.7)	DRR 0.7 (0.5 to 0.9)	DRR 2.1 (1.7 to 2.7)	DRR 2.4 (1.9 to 3)	DRR 1.9 (1.5 to 2.5)
≥60 to ≤74 years	33.1 (27.4 to 39.7)	DRR 0.5 (0.4 to 0.7)	DRR 1.4 (1.1 to 1.7)	DRR 1.9 (1.5 to 2.4)	DRR 2.3 (1.9 to 2.9)
≥75 years	86.5 (72.6 to 102.4)	DRR 0.4 (0.3 to 0.6)	DRR 1.5 (1.2 to 1.9)	DRR 1.6 (1.3 to 2)	DRR 2.3 (1.9 to 2.8)
